# Lavender oil as eco-friendly alternative to protect wood against termites without negative effect on wood properties

**DOI:** 10.1038/s41598-022-05959-5

**Published:** 2022-02-03

**Authors:** Kristýna Šimůnková, Štěpán Hýsek, Ladislav Reinprecht, Jan Šobotník, Tereza Lišková, Miloš Pánek

**Affiliations:** 1 Faculty of Forestry and Wood Sciences, Czech University of Life Sciences, Kamýcká 129, 165 00 Prague 6, Czech Republic; 2 Faculty of Wood Sciences and Technology, Technical University in Zvolen, Masarykova 24, 960 53 Zvolen, Slovak Republic; 3 Faculty of Tropical AgriSciences, Czech University of Life Sciences, Kamýcká 129, 165 00 Prague 6, Czech Republic

**Keywords:** Engineering, Materials science

## Abstract

Timber suffers from various biological damages. Recent efforts aim on nature-friendly sustainable technologies of wood protection to replace classical synthetic agents having usually negative impact on many non-target organisms including man. This research investigated the biocidal effectiveness of lavender oil (LO) in protecting the Norway spruce (*Picea abies*) wood against the termites *Reticulitermes flavipes* and the brown-rot fungus *Rhodonia placenta*. Following, selected physical characteristics of spruce wood treated with LO were evaluated: colour changes, roughness, surface wetting with water and surface free energy (SFE). Experiments showed that LO increased the resistance of spruce wood to termites nearly to the level of its treatment with commercial biocide based on trivalent boron and quaternary ammonium salt. The additional hydrophobic treatment of wood ensured its full termite-resistance even after artificial weathering in Xenotest and leaching in water according to EN 84, respectively. It shows a high potential of LO to protect wood against termites. Adversely, the effectiveness of 5% LO against rot was not sufficient. The colour of the oil-treated wood was preserved, its roughness increased slightly, and wetting and SFE led to a positive change, improving the adhesion of potentially applied coatings or adhesives for exterior exposures.

## Introduction

The shift from organophosphates and other toxic protectants of wood to environmental- and health-friendly alternatives poses numerous challenges on the applied research^1,2^. Many conventional preservatives, based on serious pollutants such as creosote oils, arsenic, chromium, chlorinated hydrocarbons or organometals^3^, are no longer tolerated in many countries^3–11^. New active substances need to be sought out, ideally from renewable raw materials, which have low toxicity and are not leached from the wood^12^. In contrast to a spectrum of unacceptable compounds, only a few synthetic biocides meet criteria expected from the future use. Irrespectively of the nature and origin of the biocide, additional hydrophobic coating of treated wood is usually used to improve the long-term stability^13–16^. Another promising alternative is targeted modification of the wood structure—thermally modified wood, acetylated wood, etc.^17^.

Two basic approaches, preventive and repressive, are commonly used to protect the wood against destroying organisms. The preventive methods include all measures of creating adverse conditions to stop the pests from interfering with the wood structure. The repressive methods take place after the wood damage, and should in theory be replaced by a viable preventive approach^18^. The structural protection is always the priority, and selection of a durable type of wood, insulation against moisture, and preventive treatment against a local pool of pests helps to sustain the wood for much longer. Also wood in historic buildings, furniture or artwork needs to be protected from damage^19^, with chemical or modification protection being more important when structural protection cannot be fully implemented^17,20–23^. The long-term efficacy of biocide treatments is closely related to the wood permeability and its surface free energy (SFE)^24–28^. The Norway spruce (*Picea abies*) wood, used as model material in this experiment, is widespread in Europe^15,29,30^. Number of works have dealt with increasing its durability, including its low permeability for preservatives^31–33^. It would thus be highly advantageous to ensure environmentally-acceptable protection of spruce wood against bio attacks using low-cost dipping technology, i.e. without technologically-demanding pressure impregnation as currently required^34^.

Various pests use diverse means of digesting the lignocellulose matrix of the wood^35^. While fungi rely upon fibred growth through the wood veins combined with release of digestive enzymes to the exterior leading to white-, brown- or soft-rots^36^, the wood-feeding insects, in particular beetles and termites, bore holes, weakening thus the wood profile and adequately also the mechanical properties^37^. Termites are the most serious insect pest in wood in subtropical and tropical areas^38,39^. Some 10% of roughly 3000 species of termites are known to cause a damage, with only a dozen of them being invasive pests of timber^40^. Termites are known for their voracious habits causing immense losses on properties, deciphered to 40 billion USD annually^41^. While termites are efficient decomposers of organic matter irrespectively of a stage of decomposition^42,43^, two ecological groups are responsible on the wood damage: dry-wood termites (Blattodea: Kalotermitidae: *Cryptotermes* mostly) and subterranean termites (Blattodea: Rhinotermitidae: *Coptotermes*, *Reticulitermes*). While dry-wood termites colonise wood items through the air and are not dependent on external water sources, they live unseen until the structure losses its integrity. On the other hand, the subterranean termites live in large populous colonies—living outside of the house, and can eventually recruit stunning mass of workers feeding on the wood per more than 100 kg a year in mature colony^41,44^.

The plant’s essential oils (EOs) are rich in a broad spectrum of molecule species (many terpenes, carbohydrates, alcohols, ethers, aldehydes and ketones^45–47^), and are recognized beneficial since the dawn of people^20,48–51^. It is thus not surprising that several extracts showed impressive wood-protecting abilities, and receive the attention from the applied sciences^50,52–54^. Many EOs reduce populations of bacteria, viruses, fungi, insects and plants, whilst many others having a strong positive effect on human health^49,55–57^. Some of them show effectiveness not only against fungi, but also against termite attack as wood preservatives^46^. Lavender (*Lavandula* sp.) belongs to the most common decorative plants in Mediterranean^58^. Production of lavender oil (LO) reaches 1500 tons a year. This oils is used mainly in cosmetics and medicine^49,59–64^. At the same time, the potential of LO in wood protection was not much studied so far (but see^50,52,65^).

To fill this gap, we conducted well-designed study of one natural compound containing LO to one commercial biocide Bochemit QB based on boric acid and alkylbenzyldimethylammonium chloride used for the protection of the Norway spruce mature wood. For these two formulations, used alone or with an additional hydrophobic substance, was tested their efficiency against two detrimental pests—the termites *Reticulitermes flavipes* and the brown-rot fungus *Rhodonia placenta*, as well as their effect on selected physical properties of wood.

## Materials and methods

### Wood samples

Samples of the Norway spruce (*Picea abies* (L.) Karst.) mature wood, with an average density of 412 kg m^−3^ and dimensions of 50 × 25 × 15 mm, were used for testing the resistance to decaying fungi according to EN 113^66^, and selected physical characteristics—colour, roughness, contact angle of wetting with distilled water, and computed surface free energy (SFE). For the termite resistance test, 50 × 15 × 10 mm samples were used, having slightly modified dimensions compared to the standard EN 118^67^ due to relatively low weight loss during short-term exposure.

Norway spruce (*Picea abies* (L.) Karst.) represent the only commercially used species from *Spruce* genera in Central Europe. It is artificially planted in commercial forests. Our testing material comes from commercial pure stands, the trees were chosen randomly, and they were free of any defects and irregularities. There was no need to identify the trees (or timber) as we have no similar species in Czech Republic and there is so no possibility of confusion. This material was obtained in compliance with all institutional, national, and international guidelines and legislation.

Prior to following treatment, wooden samples were conditioned in laboratory at a temperature of 20 °C and an air humidity of 65% to an equilibrium moisture content (MC) of 12% ± 1%.

### Substances and methods of wood treatment

The impregnation solution was made from 5% lavender (*Lavandula angustifolia* Mill. × *Lavandula latifolia* Medik.) essential oil (Yellow & Blue, Tierra Verde, Popůvky u Brna, Czech Republic), 70% ethanol and 25% distilled water. The exact identification of the plant material has not been carried out, as it is an extract from plants originating from French commercial plantations. Major effective components in lavender oil defined by producer, confirmed and certified by France Lavande (Montguers, France) as product FR-BIO-01 are: Linalool (37%), Cineole (< 12.5%), Camphor (< 11%), Limonene (1.5%), Geraniol (0.5%) and Eugenol, Coumarin, benzyl benzoate (together < 1%). The essential oil was purchased from Tierra Verde (Czech Republic) in compliance with all institutional, national, and international guidelines and legislation.

Commercial biocide “Bochemit QB” (20% boric acid, 20% alkylbenzyldimethylammonium chloride (QAC), < 9.5% 2-aminoethanol and distilled water; Bochemie Wood Care, s.r.o., Bohumín, Czech Republic) is commercial biocide—fungicidal and insecticidal water-soluble preservative—for preventive outdoor long-term protection of wood, and it was used as 20% (w/w) water-solution.

The hydrophobic substance consisted of a 6% aqueous dispersion containing 2% silane-siloxane emulsion (Lukofob DxL, Lučební závody a.s., Kolín, Czech Republic), 2% wax emulsion with glass particles about 5 μm in diameter (Horsemen concreate, Horsemen, Belgium), and 2% pure wax emulsion (Horsemen Stonecare, Horsemen, Belgium).

Both solutions were applied at room temperature by 8 h dipping. Half of treated samples was, after reconditioning to moisture content (MC) of 12 ± 2%, dipped 6 h by the hydrophobizer. The retention of solutions and active substances into the sample, as well as the labelling of the sample, are specified in Table 1.

**Table 1:** Retention of solutions and active substances into wood samples.

Set of samples	Wood species	Lavender oil (5%) Retention (kg m^−3^)		Bochemit QB (20%) Retention (kg m^−3^)			Hydrophobizer Solution retention* (kg m^−3^)
		Solution	Active LO	Solution	Boric acid	QAC	
REF-P	Pine	–	–	–	–	–	–
U-O	Spruce	–	–	–	–	–	–
U-H	Spruce	–	–	–	–	–	117 (± 20)
L-O	Spruce	121 (± 20)	6.05 (± 0.68)	–	–	–	–
L-H	Spruce	119 (± 21)	5.95 (± 0.71)	–	–	–	119 (± 19)
B-O	Spruce	–	–	119 (± 18)	4.76 (± 0.72)	4.76 (± 0.72)	–
B-H	Spruce	–	–	119 (± 18)	4.76 (± 0.72)	4.76 (± 0.72)	119 (± 21)

*Explanation of symbols: Scotch pine wood—(REF-P) is reference required by EN 113; Other symbols are valid for the Norway spruce wood: Untreated original—(U-O); Untreated, but with the additional hydrophobic substance—(U-H); Treated with the lavender oil—(L-O); Treated with the lavender oil and then with the hydrophobic substance (L-H); Treated with the Bochemit QB—(B-O); Treated with the Bochemit QB and then with the hydrophobic substance—(B-H).

Only the retention of the 6% hydrophobizer solution is given.

### Aging—leaching according to EN 84 and artificial weathering in Xenotest

The 1st group of wood samples was used for experiments without additional aging. The 2nd group of samples was subjected to a 14 days leaching procedure by EN 84^68^. The 3rd group of samples was subjected to artificial weathering in Xenotest based on standard EN 927-6^69^. The following parameters were set in the Xenotest chamber: total exposure time 320 h (2.5 h irradiation and 0.5 h of spraying in the dark in one cycle); the set radiation intensity 55 W m^−2^ (T-UV between 300–400 nm); temperature on black panel 70 °C; air temperature 45 °C, relative air humidity 30%; the total energy of the radiation that fell on the test specimens 13,210 kJ m^−2^. After aging procedures, the samples were again air-conditioned at w ≈ 12%.

### Bio-resistance to the termite *Reticulitermes flavipes* and the brown-rot fungus *Rhodonia placenta*

Six tested samples per each treatment type were subjected to *Reticulitermes flavipes* (Kollar) termite attacks according to standard EN 118^67^. Each sample was put in Petri dish (ø 90 mm) with 100 g of sand, 50 mL of distilled water and 100 termite workers. The Petri dishes were in constant dark for 6 weeks at 12 h + 30 °C and 12 h + 20 °C regime. Attack resistance was assessed in terms of mass losses (*Δm* in % scored from oven-dry wood before and after exposure).

Four wood samples, per each treatment type, were placed in 1 L Kolle glass flasks for attack by the brown-rot fungus *Rhodonia placenta* (Fr.) Niemel, K.H. Larss & Schigel, strain FPRL 280, obtained from Building Research Establishment, Garston—Watford—Herst, UK), in accordance with standard EN 113^66^. After 16 weeks, the resistance of the tested samples was evaluated on the bases of their mass losses (*Δm* in %). As reference standard were tested samples of the Scots pine (*Pinus sylvestris* L.) wood (REF-P).

### Wood surface changes in colour, wetting by water, surface free energy and roughness

Colour parameters ^70^ of spruce samples before and after lavender treatment were measured on eight samples from each series using Spectrophotometer CM-600d (Konica Minolta, Osaka, Japan) set to an observation angle of 10°, d/8 geometry, D65 light source, and the SCI method. We carried out six measurements per sample, a total of 48 per each set of samples. The colour difference according to the Euclidean distance ΔE*^70^ was calculated using the Eq. (1):

1 $$\Delta E* = \sqrt {\Delta L*^{2} + \Delta a*^{2} + \Delta b*^{2} }$$

where ΔL*, Δa*, and Δb* are relative changes in colour after lavender treatment; L* is lightness from 0 (black) to 100 (white), a* is chromaticity coordinate + (red) or − (green), and b* is chromaticity coordinate + (yellow) or − (blue).

The contact angles of distilled water (CA_water_°) were measured on goniometer Krüss DSA 30E (Krüss, Hamburg, Germany) with Krüss software (Krüss, Hamburg, Germany), and determined CA_water_° values were by ORWK model used for determination of the surface free energies (SFE in mN m^−2^). In the sessile drop method with a droplet size of 5 μL, the contact angles were measured 5 s after the drop was placed on the material (according to Petrič and Owen^71^). The measurements were performed at a room temperature of 20 °C and a relative humidity of 60%. For SFE calculation the distilled water was used as the polar liquid and the diiodomethane as non-polar fluid. Measurements were performed on a total of 8 samples from each series and a total of 48 measurements per each treatment (see Table 1).

Surface roughness before and after lavender oil treatment was studied in accordance with EN ISO 4287 (1997)^72^ and EN ISO 4288 (1996)^73^ using contact Form Talysurf Intra (Taylor Hobson, Leicester, UK). Measurements were performed for 6 samples from each series (on 4 places on each sample), i.e., totally 24 measurements in the cross direction were carried out for one series. Evaluated parameters were: R_a_—roughness average in μm; R_z_—the highest profile height in μm; R_Sm_—roughness spacing mean in μm; R_c_—average height of profile elements in μm.

### Microscopic (SEM) and elemental composition analyses

The selected surface section of the spruce samples treated with lavender oil, other treatments, controls and aged samples were analysed using a MIRA 3 scanning electron microscope (Tescan Orsay Holding, Brno, Czech Republic). The elemental compositions were examined by an energy dispersive spectroscopy system (Bruker XFlash X-ray detector, Karlsruhe, Germany, and ESPRIT 2 software).

### Statistical evaluation

Statistical analyses were performed in MS Excel 2016 and Statistica 13.2 (StatSoft, Palo Alto, CA, USA) using mean values (Mean), standard deviations (SD), whisker plots with mean values and ± 2SD, and Tukey HSD test at 95% statistical significance.

## Results and discussion

### Termite resistance

**Figure 1:**
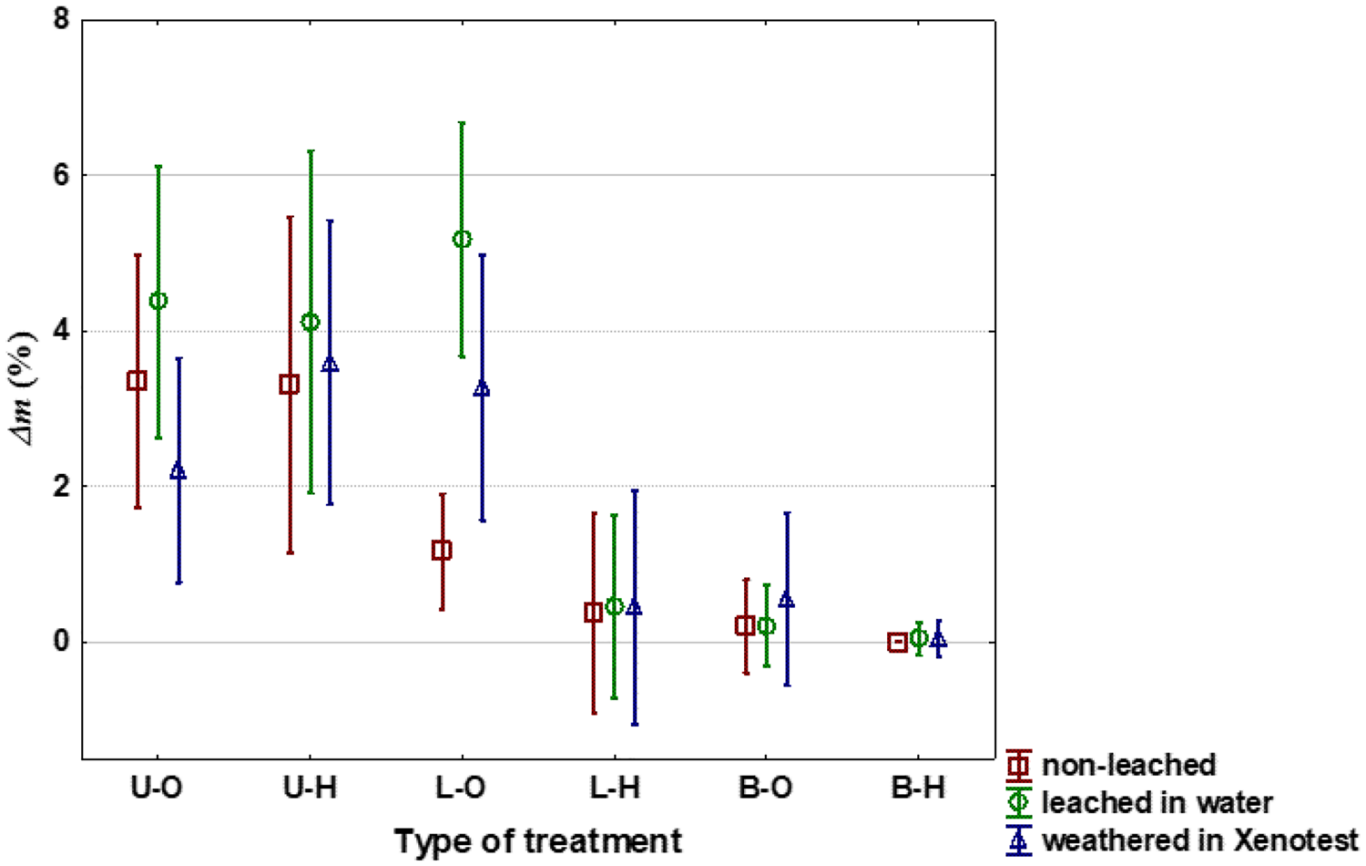
Mass losses (Δm) of the Norway spruce wood samples after 6-weeks attack by termites *Reticulitermes flavipes*. Untreated original—(U–O); Untreated, but with the additional hydrophobic substance—(U-H); Treated with the lavender oil—(L-O); Treated with the lavender oil and then with the hydrophobic substance (L-H); Treated with the Bochemit QB—(B-O); Treated with the Bochemit QB and then with the hydrophobic substance—(B-H).

Different treatments of spruce wood led to various mass losses caused by termites *R. flavipes* as presented in Fig. 1. Activity of termites was severely affected by the lavender oil treatment (Table 2). Without aging, the commercial biocide (insecticide + fungicide) Bochemit QB was only partly more effective than the lavender oil, at which no significant difference between these two substances occurred (Fig. 1). High efficacy of the lavender oil was supported by low survival of termites after feeding on treated wood (Table 2). The hydrophobic treatment alone did not protect the spruce wood from termites, but after aging procedures helped significantly to stabilise the lavender oil within the tested wood samples (Fig. 1).

**Table 2:** Survival of termites after 6 weeks of termite attack by EN 118. Untreated original—(U-O); Untreated, but with the additional hydrophobic substance—(U-H); Treated with the lavender oil—(L-O); Treated with the lavender oil and then with the hydrophobic substance (L-H); Treated with the Bochemit QB—(B-O); Treated with the Bochemit QB and then with the hydrophobic substance—(B-H).

Set of samples	Spruce Untreated—U		Spruce Lavender oil treated—L		Spruce Bochemit QB treated—B	
	U-O	U-H	L-O	L-H	B-O	B-H
**Aging type**	Survival of termites (%)					
Without aging	19.37 (20.39)	31.32 (13.82)	0.00	0.00	0.00	0.00
Leaching by EN 84	53.53 (4.94)	53.45 (11.55)	31.98 (25.91)	9.88 (11.36)	0.00	0.00
Weathering in Xenotest	41.95 (20.90)	53.13 (13.51)	53.60 (9.57)	0.00	0.00	0.00

Mean values are from 6 replicates; Numbers in parentheses are SD.

**Figure 2:**
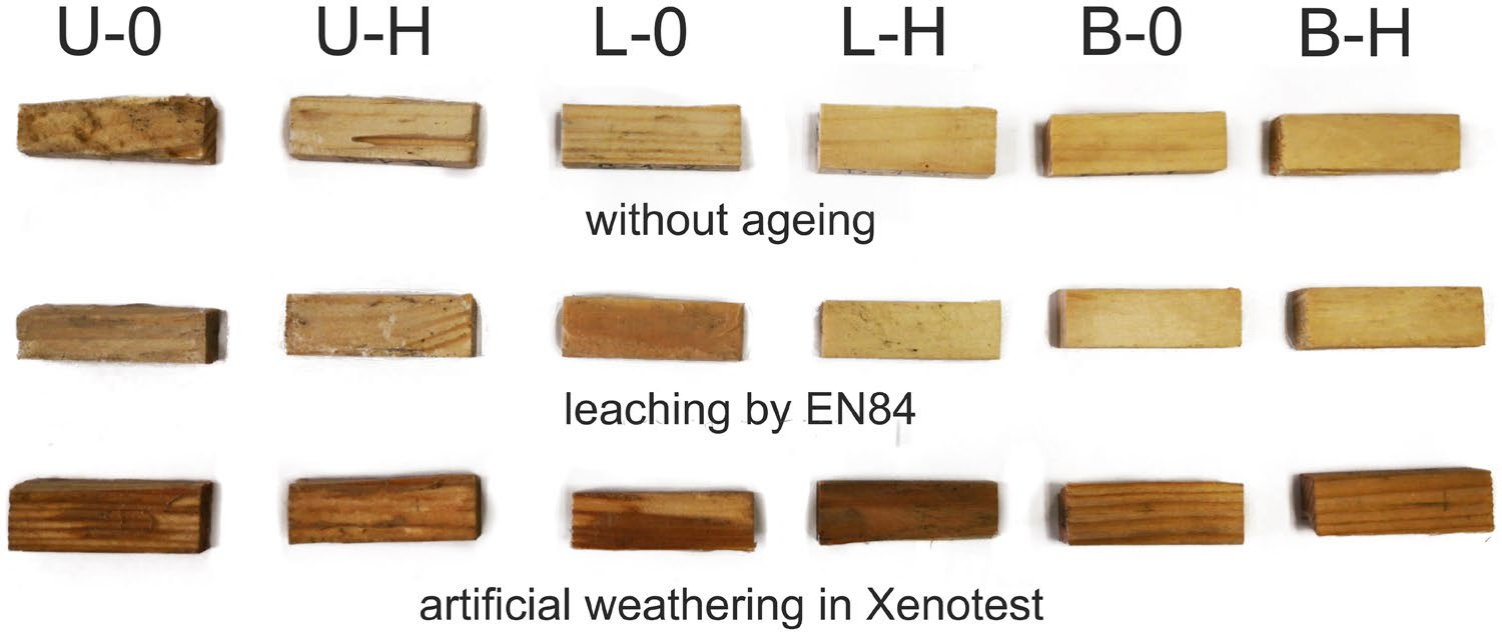
Samples demonstrating termite activity after 6 weeks of testing based on EN 118. Untreated original—(U–O); Untreated, but with the additional hydrophobic substance—(U-H); Treated with the lavender oil—(L-O); Treated with the lavender oil and then with the hydrophobic substance (L-H); Treated with the Bochemit QB—(B-O); Treated with the Bochemit QB and then with the hydrophobic substance—(B-H).

Results clearly show that lavender EO is an effective insecticide (Figs. 1 and 2), similarly to other EOs protecting the wood against various pests including termites^74–77^. Our findings are thus of prime importance as termites belong to the most feared pests of built-in wood^40,41^. Repellent effects of lavender oil can be attributed mainly to linalool, linalyl acetate, 1,8-Cineole, Camphor, Borneol, Lavandulyl acetate^47,47,76^. Work of Kartal et al.^75^ showed good efficacy of cinnamon essential oils and cinnamic derivates against subterranean termites *Coptotermes formosanus*, but only 3-weeks tests were used. More other essential oils were effective against termites, limitations for wood protection in practice are that only nutrient medium was used or no information about treatment method was disclosed^46^.

### Brown rot resistance

**Figure 3:**
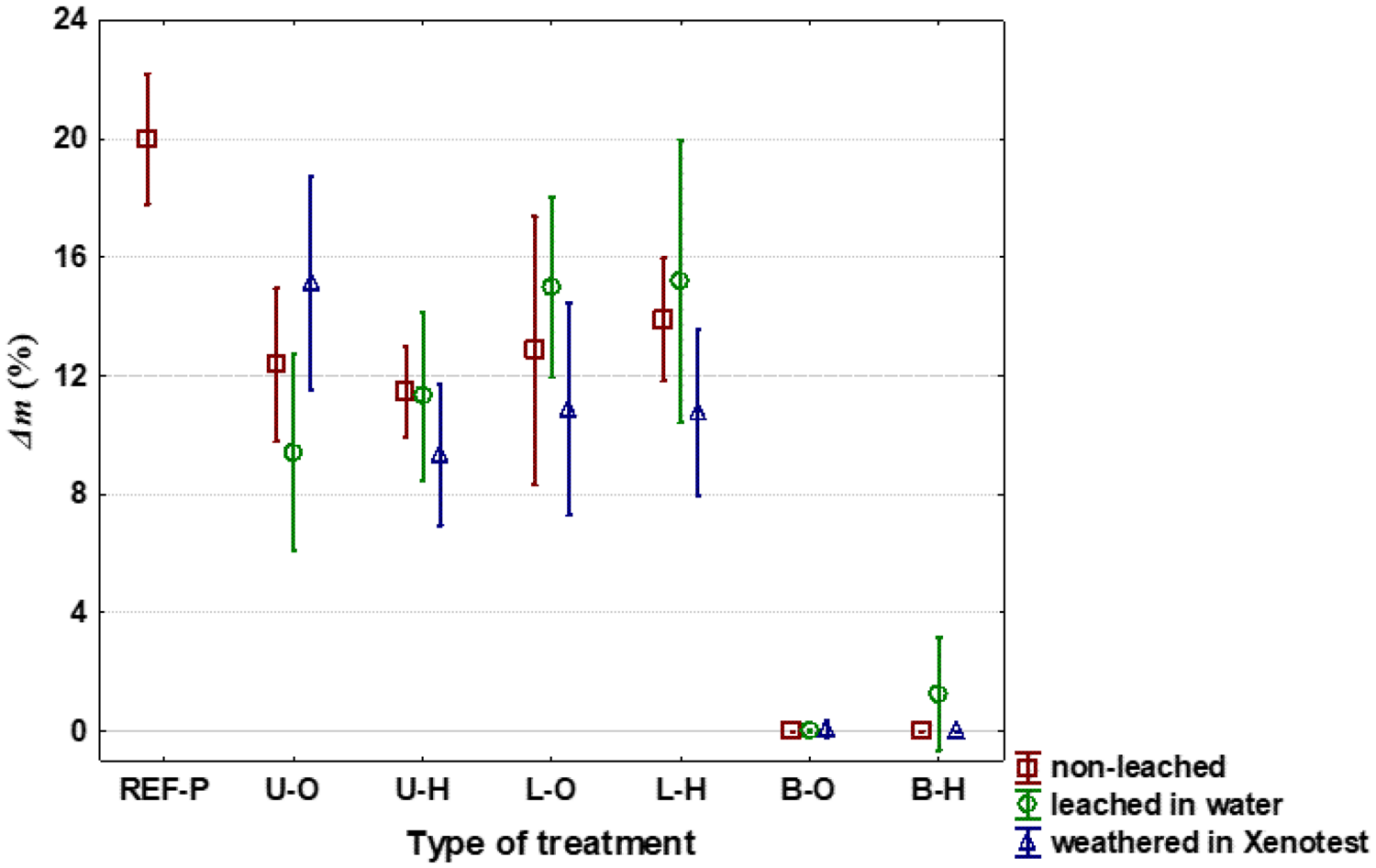
Mass losses (Δm) of wood caused with the brown-rot fungus *Rhodonia placenta* after 16-weeks. Reference—the Scotch pine wood—(REF-P); Other symbols are valid for the Norway spruce wood: Untreated original—(U–O); Untreated, but with the additional hydrophobic substance—(U-H); Treated with the lavender oil—(L-O); Treated with the lavender oil and then with the hydrophobic substance (L-H); Treated with the Bochemit QB—(B-O); Treated with the Bochemit QB and then with the hydrophobic substance—(B-H).

Lavender oil, alone or in combination with the hydrophobic substance, did not improve the spruce wood resistance against the brown-rot fungus *R. placenta* (Fig. 3). In fact, we used lower, economically acceptable 5% concentration of the lavender EO, without or in combination with hydrophobizer. On the other hand, in previous experiments higher concentrations of lavender EO (10% or more) showed at least its partial effectiveness against decaying fungi and growth of moulds^50,52,78^.

Using of vegetable oils as a solvent medium for lavender EOs can be a sound alternative thanks to hydrophobic and repellent effect of this oil itself^52,79,80^. Another possibility is using only the most active substances presented in lavender EOs, natural or synthetic. Using of natural products or their derivates as wood preservatives was shown and reviewed in more works^46,50,52,75^. Perspective seems to be mainly various extractives from cinnamon with good efficacy not only against fungi, but also against termite attack^46,75^.

### Changes in surface properties

The eventual spread of lavender oil treatment depends, apart from the wood protection itself, also on the change in wood properties. The colour parameters of spruce wood (L-O) did not change due to lavender oil impregnation: L* = 84.22 (SD = 2.24), a* = 3.43 (SD = 1.03), b* = 22.92 (SD = 1.04), compared to wood without any treatment: L* = 82.76 (SD = 0.99), a* = 3.94 (SD = 0.41), b* = 23.63 (SD = 0.58) (ΔE* = 1.70)—note that colour changes smaller than ΔE* = 3 are not recognized by a man^81^. The surface characteristics of wetting with water and SFE of lavender-treated wood have significantly improved, especially due to potential use paints and/or adhesives (Table 3). Changes of this magnitude can contribute to a significant increase in the adhesion of additional coatings or glues treatment^82,83^.

**Table 3:** CA° and SFE of Norway spruce wood without and with lavender treatment.

	CA_water_°	SFE (mN m^−2^)	SFE disperse (mN m^−2^)	SFE polar (mN m^−2^)
Untreated spruce (U-O)	105.56 (15.57)	30.48 (7.45)	30.47 (7.10)	0.01 (0.36)
Lavender treated spruce (L-O)	90.02 (6.55)	47.27 (3.16)	46.88 (2.39)	0.38 (0.77)

Mean values are from 8 replicates; Numbers in parentheses are SD.

Water repelency effect of the hydrophobic treatment was not significantly altered after artificial aging, when CA_water_° without aging (1st group) was 124.3° (SD = 13.9°), after leaching in water (2nd group) according to EN 84 was 90.9° (SD = 21.28°) and after artificial weathering in Xenotest (3rd group) was 113.15° (SD = 16.98°).

The roughness of the treated samples increased slightly, due to the bending of the wood fibres after dipping in the lavender solution^84^, but the change was only mild (Table 4).

**Table 4:** Measured roughness of spruce wood without and with lavender treatment.

Set of samples	R_a_	R_z_	R_SM_	R_c_
Untreated spruce (U-O)	7.30 (1.79)	51.97 (9.43)	430.77 (92.13)	31.52 (8.93)
Lavender treated spruce (L-O)	9,49 (2.48)	63.11 (12.70)	391.33 (113.93)	45.32 (9.51)

Mean values are from 24 measurements; Numbers in parentheses are SD.

**Figure 4:**
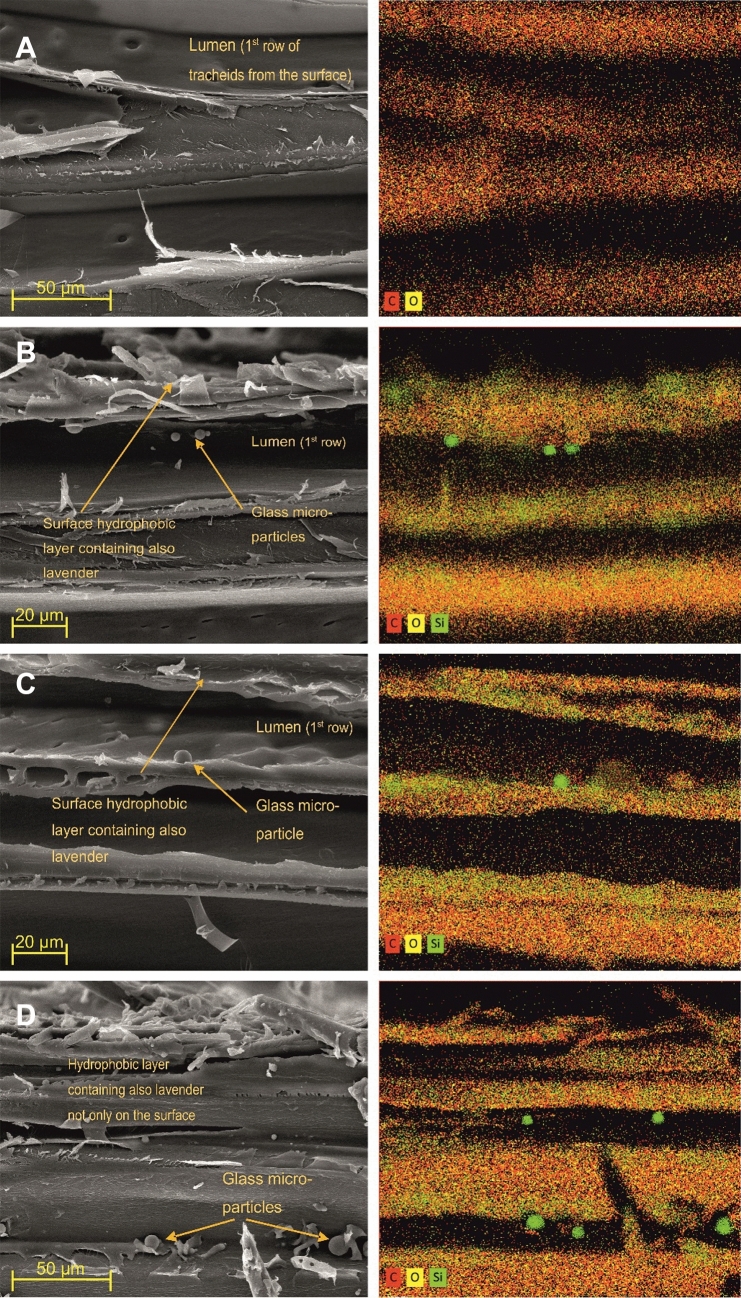
SEM micrograph (left) and the elemental composition analyses focused on Si (right) of the surface layer of spruce heartwood treated with lavender oil and a hydrophobic substance. The presence of higher concentrations of silane-siloxane compounds in cell wall structure and glass micro-particles on the surface and lumen of tracheid is shown—also after accelerated aging. (**A**) Lavender oil spruce wood treatment without aging and without hydrophobic layer 7; (**B**) Lavender oil treatment withou aging and with hydrophobic layer; (**C**) Lavender oil treatment with hydrophobic layer after leaching by water in accordance with EN 84; (**D**) Lavender oil treatment with hydrophobic layer after artificial weathering in Xenotest.

SEM and elemental analysis showed preservation of the Si-containing hydrophobic layer in silane-siloxanes and glass particles even after artificial accelerated aging (Fig. 4), what explains the above-mentioned results on CA°water measurements (Table 3).

The treatment with lavender EO did not significantly change the spruce wood appearance and roughness, respectively. At the same time, the wettability and SFE were positively affected, what make possibility for improving the adhesion of subsequently applied coatings and adhesive layers. Our results show that while strongly diluted lavender EO is extremely effective against wood-feeding insects “such as termites”, only much higher concentrations might be effective for wood protection against wood-decaying fungi^52,85^. This is an important issue when considering lavender oil use on a large industrial scale, and the cost-effectiveness and ecological impacts should be carefully weighted in the future works. Lavender EO treatment can be fully recommended as a harmless insecticide in products that come into contact with food (e.g. packing material), art objects, or for products intended for children. It can also be recommended for a short-term protection of wood or wood-based products during transport, where short-term repellence is sufficient^86,87^. Use as an eco-friendly sterilization agent against termite appears possible because lavender EO oil is highly volatile and the survival of termites in its presence documented in this research was very low (see Table 2). The most promising results comprise combination of lavender EO and hydrophobic treatment (Fig. 1), which substantially improve the wood durability outdoors, including protection against termites, i.e., the most important wood pests in warmer regions. It was shown that a simple technology of long-term dipping is sufficient even for the extremely difficult-to-permeate Norway spruce wood^31^, which opens new avenues for applied research in wood protection.

## Conclusions

This research showed a very high efficiency of highly-diluted (5%) lavender oil applied by simple dipping technology to protect spruce wood against attacks by *R. flavipes* termites. This is a very important finding as spruce wood belongs to the most common woods on the global scale, notoriously known for its low permeability to insecticides. The full protection can be achieved by a simple technology without expensive and sophisticated technologies, such as vacuum-overpressure. Lavender oil itself leaches from wood easily, however, the additional application of the hydrophobic treatment by a mixture of waxes, silane-siloxanes and glass microparticles in aqueous solution significantly increases the resistance of treated wood to termites, surprisingly to a degree ensured by toxic commercial insecticides. Unfortunately, used cost-wise friendly dilution of lavender oil was not sufficient to protect the wood against the brown-rot fungus *R. placenta*, irrespectively of surface hydrophobization. A colour change of spruce wood treated by lavender oil was negligible, however, a slight increase in the wood roughness accompany the treatment application. Other surface characteristics of spruce wood, such as wetting with water and surface free energy, have improved after lavender oil treatment, and this improve the adhesion of eventual applied coatings or glues.
